# PEMPHIGUS: ACTIVE OR INACTIVE?

**DOI:** 10.4103/0019-5154.53173

**Published:** 2009

**Authors:** Jignesh B Vaishnani, Sanjay S Bosamiya

**Affiliations:** *From the Department of Dermatology, Surat Municipal Institute of Medical Education and Research, Umarwada, Surat-10; Gujarat, India. E-mail: jigsmimer@yahoo.co.in*

Sir,

Pemphigus is a collective term for a group of chronic bullous dermatoses characterized by an intraepidermal cleft and circulating antibodies against epithelial cell surface. To date, there are no objective criteria to map the disease activity and severity. Various studies have given different and confusing definitions for remission and control of the disease. Also, there are no universally accepted treatment regimens.[1,2]

The study of activity and inactivity of the disease is important. Pemphigus is a chronic disorder with remission and exacerbation, even when patients are on treatment. Systemic steroids alone or in combination with other immunosuppressive drugs are the main stay of treatment. These drugs have to be given on long-term basis with considerable side effects, for example, osteoporosis, avascular necrosis, HPA-axis suppression, cataract, cushingoid features, myopathy, mood changes, and immunosuppression. Therefore, a system is required to monitor disease activity and inactivity so as to lower the dosage of the drug(s) and eventually withdraw and reintroduce when there is a relapse or activity reappear. Though no system is available to date that allows the withdrawal of drugs various clinical and laboratory monitoring may allow the lowering of dosage. In recent years dexamethsone cyclophosphamide in pulse doses is the treatment of choice for pemphigus. The therapy has claimed to induce remission for more than 10 years in many patients. This article is aimed at discussing the currently available clinical and laboratory systems for monitoring of pemphigus disease activity and inactivity.

The formation of new blisters indicates active stage of the disease. The considerable clinical activity usually correlates with circulating antibodies titer and antibodies in skin. Presence of new blisters in patients on treatment call for increase in dose of steroid.[3–5] After rupture of pemphigus blisters the erosions formed show no tendency to heal spontaneously and grow by confluence.[6] During active stage when lateral pressure is applied on the blister it self or perilesional skin or normal appearing skin result in removal of upper layer of epidermis known as Nikolsky's sign. Two types of Nikolsky's sign are described – wet Nikolsky's sign in which after separation of epidermis the base of skin is moist, glistening, and exudative, and dry Nikolsky's if the base of eroded skin is relatively dry. Active disease is readily appreciated by presence of a wet Nikolsky's sign, while a dry Nikolsky's sign indicates re-epithialization beneath a remnant blister top.[7] Hameed *et al*., describe a microscopic Nikolsky's sign in which tangential pressure given to an area before a biopsy produces changes in epidermis that are diagnostic of pemphigus vulgaris if in suprabasal level and pemphigus foliaceous if in intraepidermal. This is a useful sign in patients with old/infected blisters, no fresh/intact blisters, or when no facility of immunofluorescence is available in the hospital.[8]

Oral lesions, despite presenting early, may last to clear.[9,10] In addition to erosions and ulcers, extensive vegetating granulation arise from mucosa of oral cavity, which on histopathology, reveals granulation and a few acantholytic cells attached to it.[11] Yesudian *et al*.,[12] noted when healing eventually occurs in pemphigus (remission) some patients show seborrheic-keratosis-like lesions, which on histopathology, show intraepidermal cleft and few acantholytic cells within cleft; direct immunofluorescence (DIF) of such lesions show positive DIF staining indicative of disease activity. Indirect immunofluorescence (IIF) was found to be negative.

How to grade severity of the disease? Frenandes *et al*.,[10] noted that pemphigus presents with two grades of severity, the severe type is associated with rapid evaluation and extensive body involvement and marked toxemia. The moderate type is associated with slow evaluation, less generalized eruption, and low or no toxemia. Lesions on palm and sole occur late in the course of disease and carry a grave prognosis. Agrawal *et al*.,[13] suggest a scoring system in all patients with cutaneous and mucosal involvement for clinical severity and progress of the disease. Agarwal *et al*.*,*[14] report three cases wherein there was exacerbation of pemphigus preceding the appearance of pruritus. Pruritus as a symptom was also noted by Arya *et al*.[15]

IIF[16,17] may be used to conform the diagnosis by demonstrating circulating IgG antibodies in 90% of patients. Titer generally correlates with degree of disease activity. Patients in clinical remission with persistent high antibody titer are more likely to develop a relapse on cessation of therapy or rapid lowering of dose of steroids. Repetitive negative titer may allow withdrawal of the drug or allow minimal dosage schedule. Apart from error in substrate or reagent, an IIF could be false negative due to prozone phenomenon. False-positive results can be seen in extensive burns, penicillin reactions, and even in normal persons. Further sensitivity is lost while using different substrates and different laboratories each time. Many studies have shown that clinical activity and duration of treatment on IIF titer were not appropriate; further, IIF is not as sensitive as DIF to detect small amounts of IgG circulating antibody.[12]

In patients of clinical remission the decision about further reduction in dose of drug can be very difficult. Clinical criteria and IIF which are not sensitive enough to detect low levels of circulating antibody so cannot be used.[2,12] In state of remission, DIF[17–21] is more sensitive and small amounts of IgG deposited in epidermis can be easily detected, while the same amount cannot be detected by IIF. Positive IF (both IIF and DIF) in state of remission indicates an immunological activity of the disease and therefore treatment should be continued. IF should be performed every 2–4 weeks until there is remission of the disease and every 1–6 monthly thereafter.

Desmoglein ELISA[19,22] is a sensitive and specific test to detect auto-antibody against desmoglein (DsG) proteins, both DsG-1 and DsG-3. Quantitative data from ELISA on DsG-1 and DsG-3 show a relationship between DsG-1 antibody and skin severity and between DsG-3 antibody level and oral disease severity. Further, pemphigus vulgaris entirely limited to the mucosal surface was seen only in DsG-3, while additional DsG-1 (DsG-1 plus DsG-3) predicts cutaneous involvement in addition to mucosal involvement. Also, transition between pemphigus vulgaris and pemphigus foliaceous correlates with the changes of autoantibodies against either DsG-1 or DsG-3.[23,24] Nguyen *et al*.,[25] demonstrated non-desmoglein antibody from patients with pemphigus, some of these antibodies are recognized by keratenocytes cholinergic receptor. This antibody appears to induce inactivation of keratenocytes cholinergic receptor, disrupt the normal cell adhesion, and thus leads to acantholysis.

Beta-glucuronidase[26]: This lysosomal enzyme level is found to be raised during active disease stage and it is observed that there is a significant decrease in this enzyme activity, when lysosomal membrane stability is maintained by adequate dosage of steroids. So beta-glucuronidase can be used as a biochemical marker in assessing the response to treatment as well as maintenance treatment with steroids.

Activity in pemphigus can be defined by: 1) presence of new blisters, 2) spontaneous peripheral extension of existing blisters, 3) positive Nikolsky's sign on lesion, perilesional, or normal appearing skin, 4) demonstration of circulating antibodies against cell adhesion molecules and/or non-desmoglein protein antigen, e.g., keratinocytes cholinergic receptors, 5) presence of IgG antibodies in epidermis demonstrated by DIF, even in clinically normal appearing skin.
